# Thoracoscopic surgery for lung emphysema using an infrared camera

**DOI:** 10.1186/1749-8090-8-134

**Published:** 2013-05-24

**Authors:** Keitaro Matsumoto, Isao Sano, Hideki Taniguchi, Naoya Yamasaki, Tomoshi Tsuchiya, Takuro Miyazaki, Koichi Tomoshige, Takeshi Nagayasu

**Affiliations:** 1 Division of Surgical Oncology, Department of Translational Medical Sciences, Nagasaki University Graduate School of Biomedical Sciences, Nagasaki, Japan; 2 Department of Surgery, Japanese Red Cross Nagasaki Genbaku Hospital, Nagasaki, Japan

**Keywords:** Emphysema, VATS, Segmentectomy, Indocyanine green, Infrared camera

## Abstract

Localized emphysema is difficult to detect on normal thoracoscopy. Indocyanine green (ICG) was used to precisely delineate an emphysematous lesion using an infrared camera system in a 75-year-old woman with a large emphysematous lesion in the right lower lobe. Due to repeated infections of the emphysematous lesion, right basal segmentectomy for localized lung emphysema was performed. During surgery, ICG (0.5 mg/kg) was injected intravenously, and the emphysematous lesion was detected as a fluorescence defect. This method could be used for precise resection of large emphysematous lesions because it permits clear detection with a small amount of ICG.

## Background

In some cases of localized emphysema, it is difficult to detect the line between normal lung and the emphysematous lesion. In particular, in infected lesions, accurate detection of the line is very important to remove the entire lesion to completely eradicate infection. An infrared camera was used to precisely delineate an emphysematous lesion using an appropriate amount of indocyanine green (ICG). This method has already been used in hepatectomy by injection of ICG to identify the border of a liver segment [1] and a lung segment [2]. The case of a patient who underwent segmentectomy for a large emphysematous lesion using ICG detected by an infrared camera system (Photodynamic Eye: PDE-neo™, Hamamatsu Photonics KK, Shizuoka, Japan) is reported.

## Case presentation

**Figure 1:**
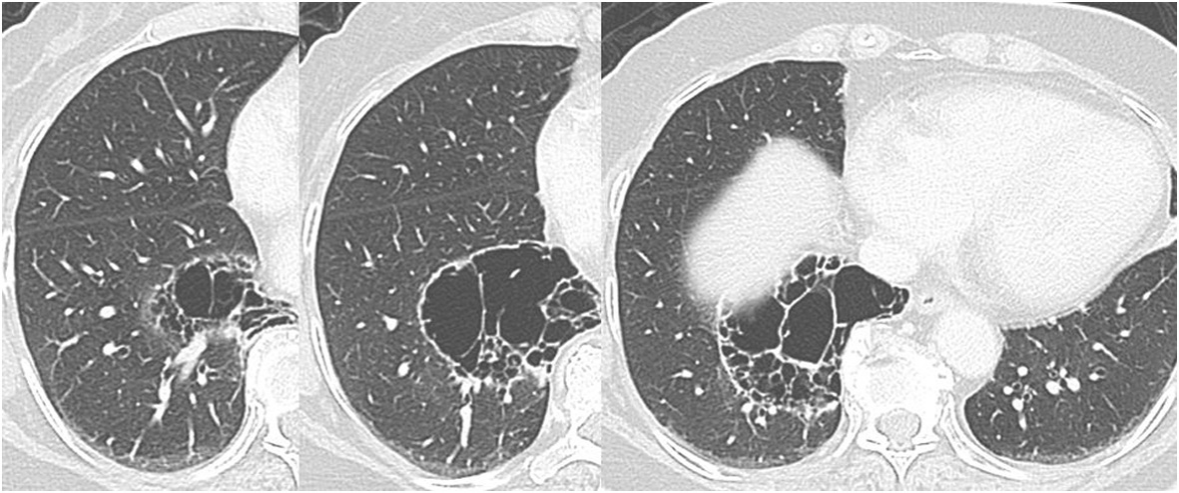
**Chest computed tomographic images show bullous emphysema in the right lower lobe.** There is no aberrant artery and no communication with the bronchus.

A 75-year-old woman was referred to our department with a diagnosis of pneumonia with a high fever. She had no significant medical history except for diabetes mellitus, and no abnormality on screening chest X-ray had ever been detected. A computed tomography (CT) scan showed fluid collection in a large emphysematous lesion. At first, pulmonary sequestration of the right lower lobe was suspected, but it was eventually diagnosed as pulmonary bullous emphysema because there was no aberrant artery from the aorta on enhanced computed tomography. The patient was treated with antibiotics for infected bullous emphysema. There was no clear communication between the bronchus and the bulla on bronchoscopy, but the presence of infection suggested the possibility of a communication. After the infection was cured, the localized emphysema had not decreased in size, and she continued to have repeated infection in the bulla. Therefore, it was decided that surgery to completely remove the bullous emphysematous lesion was needed. The large bullous lesion was located by CT scan from segment 9 to segment 10 in the right lower lobe, and it extended longitudinally to the border between segments 6 and 10 (Figure 1).

**Figure 2:**
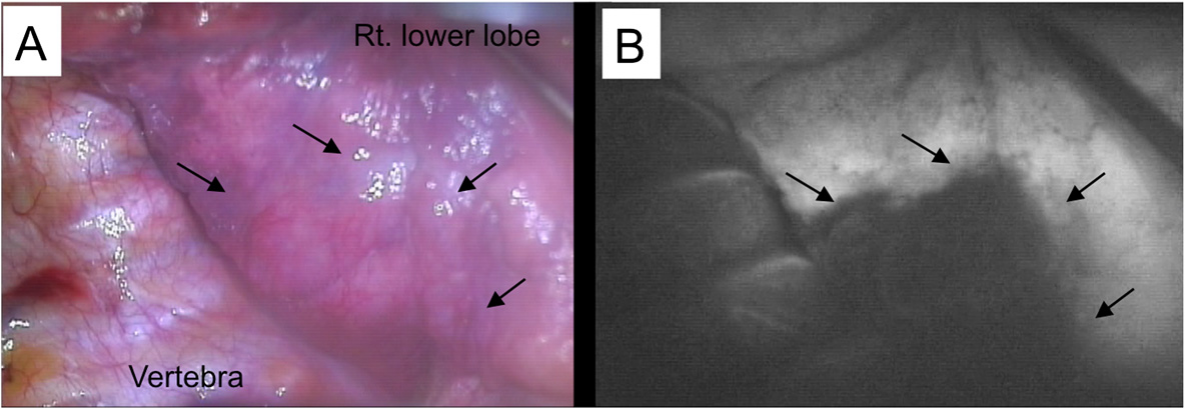
**Intraoperative images.** Images under normal light (**A**) and infrared light with indocyanine green injection (**B**). Bullous emphysema is black, whereas the normal lung tissue is white (**B**). The arrows indicate the emphysematous lesion.

Video-assisted thoracoscopic surgery (VATS) was performed, and the bullous lesion was seen both by the scope and directly by the naked eye. However, the border of the bullous lesion was not clear in the deflated lung. ICG (0.5 mg/kg) was injected into a right arm vein, and the lung was observed using the PDE imaging system through the 8-cm thoracotomy site. About 8 seconds after injection, ICG entered the lung, and the bullous lesion was detected as a defect of fluorescence contrasting with the normal lung fluorescence. The contrast remained for 30 seconds, and the ICG reached the bullous area, which was visualized as white (Figure 2). While the contrast was present, the border between normal lung and the bullous lesion was marked by electric cautery. During the marking, a scope from a 10-mm port assisted in visualizing the lung because the PDE system shows a black and white view without normal light support. It appeared that the bullous emphysematous lesion could be resected completely by basal segmentectomy, and this was performed. There were no complications postoperatively, and the patient was discharged 10 days postoperatively. At follow-up 12 months after surgery, the patient had no infections and no bullous lesions in the lung. The patient was provided informed consent to use ICG and the Ethics Review Board on Clinical Research of Japanese Red Cross Nagasaki Genbaku Hospital approved the study protocol (#175).

## Conclusions

A localized pulmonary bulla is sometimes difficult to detect clearly on thoracoscopy, especially in a deflated lung. VATS has already been accepted for wedge resection of lung bullae or emphysema and partially for lobectomy or segmentectomy of lung cancer or infectious diseases of the lung [3,4]. Under VATS, inflation of the lung makes the operation difficult because a large part of the chest cavity is occupied. To address this issue, Gotoh et al. used ICG to detect bullae of pneumothorax by infrared thoracoscopy [5]. Their infrared camera was very useful because it was a two-color infrared camera that showed the tissue with ICG as blue and that without ICG as white. However, they used ICG 3.0 mg/kg, which is much more than the volume of ICG used for liver function testing [5]. According to toxicity studies, a 5.0 mg/kg ICG intravenous injection is safe and tolerable [6], and another report described it as safe enough, as there were only three (0.15%) mild adverse reactions, four (0.2%) moderate reactions, and one (0.05%) severe reaction in 1226 consecutive patients [7]. However, some reports noted that ICG dye has a dose-dependent toxic effect on the retina [8], and in our experience, prolonged vomiting or fever has occurred postoperatively in some cases. It is clear that a smaller volume of ICG than they used is much better.

The PDE imaging system contains a charge-coupled device camera that filters out light with a wavelength of less than 820 nm, as well as 36 light-emitting diodes with a wavelength of 760 nm. It shows clearly that the tissue with ICG is white and that without ICG is black [9]. The advantage of the PDE system is high sensitivity for ICG, which allows a lower dose of ICG (0.5 mg/kg) to be used. Since even ICG at 0.5 mg/kg can show the contrast clearly, it may be possible to reduce the ICG dose further. Another advantage of this approach includes the ability to see the border clearly as a black and white line and to be able to use normal thoracoscopy from another 10-mm port to see the normal color lung at the same time. This means that it is possible to use electrocautery to mark the border using normal thoracoscopy and the PDE system concurrently. The black and white contrast lasted for about 30 seconds, and then gradually the bullous lesion turned white; this is not long, but it is enough to see the border and mark it by electric cautery. Furthermore, because the color is lost about 15 minutes after injection, the segment line can be used one more time during cutting. On the other hand, a disadvantage is the large size of the system, having a diameter of 8 cm, which requires a large incision to be used. However, in our experience, it is possible to use at least a 4-cm incision because the part of the charged-coupled device (CCD) camera except for the infrared light-emitting diode (LED) is 3.2 cm in diameter.

In conclusion, the procedure using the PDE system with ICG injection to perform surgery for pulmonary bullous emphysema is safe and can be quickly completed, with minimal drug-related side effects. This approach is cost-effective because the PDE system can be used in other operations, and ICG is inexpensive. ICG fluorescence for lung operations promises to be useful.

## Consent

Written informed consent was obtained from the patient for publication of this Case report and any accompanying images. A copy of the written consent is available for review by the Editor-in-Chief of this journal.

## Abbreviations

CCD: Charged-coupled device; CT: Computed tomography; ICG: Indocyanine green; LED: Light-emitting diode; PDE: Photodynamic Eye; VATS: Video-assisted thoracoscopic surgery.

## Competing interests

The authors declare that they have no competing interests.

## Authors’ contribution

KM wrote the manuscript, and TN supervised the patient’s entire treatment. IS and HT participated in the surgical operation and treatment. NY, TT, TM, and KT collected the data and gave the PDE information. All authors read and approved the final manuscript.
